# Supplementary material for: A Bayesian framework for multivariate differential analysis
Source: PLoS Comput Biol. 2026 Sep 2;22(9):e1014637. doi: 10.1371/journal.pcbi.1014637 (PMC13561429; doi:10.1371/journal.pcbi.1014637)
Supplement: Supplementary file 1 [file pcbi.1014637.s001.pdf]

Supporting Information for A Bayesian Framework for Multivariate Differential Analysis

Marie Chion & Arthur Leroy

Contents

A Notations and abbreviations **2**

B Proofs **3**

B.1 Proof of Bayesian inference for Normal-Inverse-Gamma conjugated priors 3

B.2 Proof of General Bayesian framework for evaluating mean differences 5

C Sensitivity analysis for hyperparameters **8**

C.1 Sensitivity of α_0, β_0, and λ_0 8

C.2 Sensitivity of Σ_0 8

C.3 Sensitivity of ν_0 8

C.4 Joint effect of α_0 and β_0 10

C.5 Uncertainty calibration for different thresholds 10

D Real-data experiments: additional results **12**

E Simulation experiment on MNAR data **13**

A Notations and abbreviations

**Symbol**	**Meaning**
y	Observation in the univariate model.
y_n	n-th observed value in a univariate sample.
$\mathbf{y}_k$ or $y_k \in \mathbb{R}^P$	Observation vector for group/condition k in the multivariate model.
$y_{k,n}$	n-th sample in group k.
$y_{k,n}^p$	Value for peptide/feature p, sample n, group k.
μ	Mean parameter in the univariate model.
μ_k	Mean vector for group k.
μ_k^p	Mean of peptide/feature p in group k.
μ_0	Prior mean hyperparameter.
λ_0	Prior precision-like hyperparameter controlling confidence in μ_0.
σ^2	Variance in the univariate model.
Σ_k	Covariance matrix for group k.
Σ_0	Prior covariance-scale hyperparameter in the multivariate model.
α_0, β_0	Inverse-gamma prior hyperparameters for variance.
ν_0	Inverse-Wishart prior degrees-of-freedom hyperparameter.
ε	Univariate error term.
ε_k	Multivariate error term for group k.
N	Number of observations in the univariate derivations.
N_k	Number of samples in group k.
N_k^p	Number of observed values for peptide p in group k.
K	Number of groups/conditions.
P	Number of peptides/features jointly modelled.
$\bar{y}$	Sample mean in the univariate model.
$\bar{y}_k^{(d)}$	Mean vector of the d-th imputed dataset in group k.
$\bar{y}_k^{p,(0)}$	Observed-data mean for peptide p in group k.
$\mu_N, \lambda_N, \alpha_N, \beta_N$	Updated posterior hyperparameters in the univariate conjugate model.
$\mu_{k,N}^{(d)}, \lambda_{k,N}, \Sigma_{k,N}^{(d)}, \nu_{k,N}$	Updated posterior hyperparameters for group k in the multivariate algorithm.
$\hat{\sigma}^2$	Scale parameter of the marginal posterior Student distribution for μ.
$\hat{\sigma}_k^{p\,2}$	Univariate posterior scale for peptide p in group k.
$T_\nu(\cdot)$	Student t distribution with ν degrees of freedom.
$W^{-1}(\Sigma_0, \nu_0)$	Inverse-Wishart prior for covariance matrices.
$\Gamma^{-1}(\alpha_0, \beta_0)$	Inverse-gamma prior for variance.
H	Set of observed data.
$y_k^{(0)}$	Observed entries in group k.
$y_k^{(1)}$	Missing entries in group k.
$\tilde{y}_k^{(1),d}$	d-th imputed version of the missing data in group k.
$\tilde{y}_{k,n}^{(d)}$	Complete vector for sample n, group k, after d-th imputation.
D	Number of imputed datasets / imputation draws.

Symbol	Meaning
R	Number of posterior samples drawn for inference.
d_r	Random imputation index drawn in Algorithm 1.
$\hat{\mu}_k^{[r]}$	r-th posterior draw of the mean for group k.
$\hat{\mu}_\Delta^{[r]}$	r-th posterior draw of the difference between two groups.
$p(\mu_k - \mu_{k'} \mid y_k, y_{k'})$	Posterior distribution of the difference of means between groups k and k'.
μ_{diff}	Estimated mean difference, defined as $\hat{\mu}_1 - \hat{\mu}_2$.
CI_{95}	95% credible interval.
CI_{95} width	Width of the 95% credible interval.
RMSE	Root mean square error.
CIC_{95}	95% credible interval coverage.
$\mu_{p,k}^{\text{true}}$	Reconstructed reference mean for peptide p in group k.
Σ_{ref}	Baseline covariance matrix used in multivariate simulations.
N^+	Number of peptides with larger values in one group than the other in multivariate inference.
N^-	Number of peptides with lower values in one group than the other.
MAR	Missing At Random.
MNAR	Missing Not At Random.

Table A: Notation and abbreviation table.

B Proofs

B.1 Proof of Bayesian inference for Normal-Inverse-Gamma conjugated priors

Let us recall below the complete development of this derivation by identification of the analytical form (we ignore conditioning over the hyperparameters for convenience):

$$\begin{aligned}
p(\mu, \sigma^2 \mid \boldsymbol{y}) &\propto p(\boldsymbol{y} \mid \mu, \sigma^2) \times p(\mu, \sigma^2) \\
&= \left(\frac{1}{2\pi\sigma^2}\right)^{\frac{N}{2}} \exp\left(-\frac{1}{2\sigma^2}\sum_{n=1}^{N}(y_n - \mu)^2\right) \\
&\quad \times \frac{\sqrt{\lambda_0}}{\sqrt{2\pi}} \frac{\beta_0^{\alpha_0}}{\Gamma(\alpha_0)} \left(\frac{1}{\sigma^2}\right)^{\alpha_0 + \frac{3}{2}} \exp\left(-\frac{2\beta_0 + \lambda_0(\mu - \mu_0)^2}{2\sigma^2}\right) \\
&\propto \left(\frac{1}{\sigma^2}\right)^{\alpha_0 + \frac{N+3}{2}} \exp\left(\underbrace{-\frac{2\beta_0 + \lambda_0(\mu - \mu_0)^2 + \sum_{n=1}^{N}(y_n - \mu)^2}{2\sigma^2}}_{\mathcal{A}}\right).
\end{aligned}$$

Let us introduce Lemma 1 below to decompose the term $\mathcal{A}$ as desired:

Lemma 1. *Assume a set* $\boldsymbol{x_1}, \dots, \boldsymbol{x_N} \in \mathbb{R}^q$*, and note* $\bar{\boldsymbol{x}} = \frac{1}{N}\sum_{n=1}^{N} \boldsymbol{x_n}$ *the associated average vector. For any* $\boldsymbol{\mu} \in \mathbb{R}^q$*:*

$$\sum_{n=1}^{N} (\boldsymbol{x_n} - \boldsymbol{\mu})(\boldsymbol{x_n} - \boldsymbol{\mu})^\intercal = N(\bar{\boldsymbol{x}} - \boldsymbol{\mu})(\bar{\boldsymbol{x}} - \boldsymbol{\mu})^\intercal + \sum_{n=1}^{N} (\boldsymbol{x_n} - \bar{\boldsymbol{x}})(\boldsymbol{x_n} - \bar{\boldsymbol{x}})^\intercal.$$

Proof.

$$\begin{aligned}
\sum_{n=1}^{N} (\boldsymbol{x_n} - \boldsymbol{\mu})(\boldsymbol{x_n} - \boldsymbol{\mu})^\intercal &= \sum_{n=1}^{N} \boldsymbol{x_n}\boldsymbol{x_n}^\intercal + \boldsymbol{\mu}\boldsymbol{\mu}^\intercal - 2\boldsymbol{x_n}\boldsymbol{\mu}^\intercal \\
&= N\boldsymbol{\mu}\boldsymbol{\mu}^\intercal - 2N\bar{\boldsymbol{x}}\boldsymbol{\mu}^\intercal + \sum_{n=1}^{N} \boldsymbol{x_n}\boldsymbol{x_n}^\intercal \\
&= N\boldsymbol{\mu}\boldsymbol{\mu}^\intercal + N\bar{\boldsymbol{x}}\bar{\boldsymbol{x}}^\intercal + N\bar{\boldsymbol{x}}\bar{\boldsymbol{x}}^\intercal - 2N\bar{\boldsymbol{x}}\bar{\boldsymbol{x}}^\intercal - 2N\bar{\boldsymbol{x}}\boldsymbol{\mu}^\intercal + \sum_{n=1}^{N} \boldsymbol{x_n}\boldsymbol{x_n}^\intercal \\
&= N\left(\bar{\boldsymbol{x}}\bar{\boldsymbol{x}}^\intercal - \boldsymbol{\mu}\boldsymbol{\mu}^\intercal - 2\bar{\boldsymbol{x}}\boldsymbol{\mu}^\intercal\right) + \sum_{n=1}^{N} \boldsymbol{x_n}\boldsymbol{x_n}^\intercal + \bar{\boldsymbol{x}}\bar{\boldsymbol{x}}^\intercal - 2\boldsymbol{x_n}\bar{\boldsymbol{x}}^\intercal \\
&= N\left(\bar{\boldsymbol{x}} - \boldsymbol{\mu}\right)\left(\bar{\boldsymbol{x}} - \boldsymbol{\mu}\right)^\intercal + \sum_{n=1}^{N} (\boldsymbol{x_n} - \bar{\boldsymbol{x}})(\boldsymbol{x_n} - \bar{\boldsymbol{x}})^\intercal.
\end{aligned}$$

□

Applying this result in our context for $q = 1$, we obtain:

$$\begin{aligned}
\mathcal{A} &= -\frac{1}{2\sigma^2}\left(2\beta_0 + \lambda_0(\mu - \mu_0)^2 + N(\bar{y} - \mu)^2 + \sum_{n=1}^{N}(y_n - \bar{y})^2\right) \\
&= -\frac{1}{2\sigma^2}\left(2\beta_0 + \sum_{n=1}^{N}(y_n - \bar{y})^2 + (\lambda_0 + N)\mu^2 - 2\mu(N\bar{y} + \lambda_0\mu_0) + N\bar{y}^2 + \lambda_0\mu_0^2\right) \\
&= -\frac{1}{2\sigma^2}\Bigg(2\beta_0 + \sum_{n=1}^{N}(y_n - \bar{y})^2 + N\bar{y}^2 + \lambda_0\mu_0^2 \\
&\qquad + (\lambda_0 + N)\left[\mu^2 - 2\mu\frac{N\bar{y} + \lambda_0\mu_0}{\lambda_0 + N} + \left(\frac{N\bar{y} + \lambda_0\mu_0}{\lambda_0 + N}\right)^2 - \left(\frac{N\bar{y} + \lambda_0\mu_0}{\lambda_0 + N}\right)^2\right]\Bigg) \\
&= -\frac{1}{2\sigma^2}\Bigg(2\beta_0 + \sum_{n=1}^{N}(y_n - \bar{y})^2 + N\bar{y}^2 + \lambda_0\mu_0^2 - \frac{(N\bar{y} + \lambda_0\mu_0)^2}{\lambda_0 + N} \\
&\qquad + (\lambda_0 + N)\left(\mu - \frac{N\bar{y} + \lambda_0\mu_0}{\lambda_0 + N}\right)^2\Bigg) \\
&= -\frac{1}{2\sigma^2}\Bigg(2\beta_0 + \sum_{n=1}^{N}(y_n - \bar{y})^2 + \frac{(\lambda_0 + N)(N\bar{y}^2 + \lambda_0\mu_0^2) - N^2\bar{y}^2 - \lambda_0^2\mu_0^2 + 2N\bar{y}\lambda_0\mu_0}{\lambda_0 + N}
\end{aligned}$$

$$+ (\lambda_0 + N) \left(\mu - \frac{N\bar{y} + \lambda_0 \mu_0}{\lambda_0 + N} \right)^2 \Bigg)$$

$$= -\frac{1}{2\sigma^2} \left(2\beta_0 + \sum_{n=1}^{N} (y_n - \bar{y})^2 + \frac{\lambda_0 N}{\lambda_0 + N} (\bar{y} - \mu_0)^2 + (\lambda_0 + N) \left(\mu - \frac{N\bar{y} + \lambda_0 \mu_0}{\lambda_0 + N} \right)^2 \right).$$

B.2 Proof of General Bayesian framework for evaluating mean differences

Proof. For the sake of clarity, let us omit the K groups here and first consider a general case with $\boldsymbol{y}_k = \boldsymbol{y} \in \mathbb{R}^P$. Moreover, let us focus on only one imputed dataset and maintain the notation $\tilde{\boldsymbol{y}}_1^{(d)}, \dots, \tilde{\boldsymbol{y}}_N^{(d)} = \boldsymbol{y}_1, \dots, \boldsymbol{y}_N$ for convenience. From the hypotheses of the model, we can derive $\mathcal{L}$, the posterior log-PDF over $(\boldsymbol{\mu}, \boldsymbol{\Sigma})$, following the same idea as for the univariate case presented in **??**:

$$\begin{aligned}
\mathcal{L} &= \log p(\boldsymbol{\mu}, \boldsymbol{\Sigma} \mid \boldsymbol{y}_1, \dots, \boldsymbol{y}_N) \\
&= \log \underbrace{p(\boldsymbol{y}_1, \dots, \boldsymbol{y}_N \mid \boldsymbol{\mu}, \boldsymbol{\Sigma})}_{\mathcal{N}(\boldsymbol{\mu}, \boldsymbol{\Sigma})} + \log \underbrace{p(\boldsymbol{\mu}, \boldsymbol{\Sigma})}_{\mathcal{NW}^{-1}(\boldsymbol{\mu}_0, \lambda_0, \boldsymbol{\Sigma}_0, \nu_0)} + C_1 \\
&= -\frac{N}{2} \log |\boldsymbol{\Sigma}| - \frac{1}{2} \left(\sum_{n=1}^{N} (\boldsymbol{y}_n - \boldsymbol{\mu})^\intercal \boldsymbol{\Sigma}^{-1} (\boldsymbol{y}_n - \boldsymbol{\mu}) \right) \\
&\quad - \frac{\nu_0 + P + 2}{2} \log |\boldsymbol{\Sigma}| - \frac{1}{2} \left(\operatorname{tr}\left(\boldsymbol{\Sigma}_0 \boldsymbol{\Sigma}^{-1} \right) - \frac{\lambda_0}{2} (\boldsymbol{\mu} - \boldsymbol{\mu}_0)^\intercal \boldsymbol{\Sigma}^{-1} (\boldsymbol{\mu} - \boldsymbol{\mu}_0) \right) + C_2 \\
&= -\frac{1}{2} \Bigg[(\nu_0 + P + 2 + N) \log |\boldsymbol{\Sigma}| + \operatorname{tr}\left(\boldsymbol{\Sigma}_0 \boldsymbol{\Sigma}^{-1} \right) \\
&\quad + \sum_{n=1}^{N} \operatorname{tr}\left((\boldsymbol{y}_n - \boldsymbol{\mu})^{\mathrm{T}} \boldsymbol{\Sigma}^{-1} (\boldsymbol{y}_n - \boldsymbol{\mu}) \right) + \operatorname{tr}\left(\lambda_0 (\boldsymbol{\mu} - \boldsymbol{\mu}_0)^\intercal \boldsymbol{\Sigma}^{-1} (\boldsymbol{\mu} - \boldsymbol{\mu}_0) \right) \Bigg] + C_2 \\
&= -\frac{1}{2} \Bigg[(\nu_0 + P + 2 + N) \log |\boldsymbol{\Sigma}| + \operatorname{tr}\Bigg(\boldsymbol{\Sigma}^{-1} \Big\{ \boldsymbol{\Sigma}_0 + \lambda_0 (\boldsymbol{\mu} - \boldsymbol{\mu}_0)(\boldsymbol{\mu} - \boldsymbol{\mu}_0)^\intercal \\
&\quad \underbrace{+ N(\bar{\boldsymbol{y}} - \boldsymbol{\mu})(\bar{\boldsymbol{y}} - \boldsymbol{\mu})^\intercal + \sum_{n=1}^{N} (\boldsymbol{y}_n - \bar{\boldsymbol{y}})(\boldsymbol{y}_n - \bar{\boldsymbol{y}})^\intercal}_{\text{Lemma 1}} \Big\} \Bigg) \Bigg] + C_2 \\
&= -\frac{1}{2} \Bigg[(\nu_0 + P + 2 + N) \log |\boldsymbol{\Sigma}| + \operatorname{tr}\Bigg(\boldsymbol{\Sigma}^{-1} \Big\{ \boldsymbol{\Sigma}_0 + \sum_{n=1}^{N} (\boldsymbol{y}_n - \bar{\boldsymbol{y}})(\boldsymbol{y}_n - \bar{\boldsymbol{y}})^\intercal \\
&\quad + (N + \lambda_0) \boldsymbol{\mu} \boldsymbol{\mu}^\intercal - \boldsymbol{\mu} \left(N \bar{\boldsymbol{y}}^\intercal + \lambda_0 \boldsymbol{\mu}_0^\intercal \right) - (\lambda_0 \boldsymbol{\mu}_0 + N \bar{\boldsymbol{y}}) \boldsymbol{\mu}^\intercal + \lambda_0 \boldsymbol{\mu}_0 \boldsymbol{\mu}_0^\intercal + N \bar{\boldsymbol{y}} \bar{\boldsymbol{y}}^\intercal \Big\} \Bigg) \Bigg] + C_2 \\
&= -\frac{1}{2} \Bigg[(\nu_0 + P + 2 + N) \log |\boldsymbol{\Sigma}|
\end{aligned}$$

$$
+ \operatorname{tr}\Bigg(\boldsymbol{\Sigma}^{-1} \Big\{ \boldsymbol{\Sigma}_0 + \sum_{n=1}^{N} (\boldsymbol{y}_n - \bar{\boldsymbol{y}})(\boldsymbol{y}_n - \bar{\boldsymbol{y}})^\intercal + \frac{N\lambda_0}{N+\lambda_0} (\bar{\boldsymbol{y}} - \boldsymbol{\mu}_0)(\bar{\boldsymbol{y}} - \boldsymbol{\mu}_0)^\intercal
$$

$$
+ (N+\lambda_0) \left(\boldsymbol{\mu} - \frac{N\bar{\boldsymbol{y}} + \lambda_0 \boldsymbol{\mu}_0}{N+\lambda_0} \right) \left(\boldsymbol{\mu} - \frac{N\bar{\boldsymbol{y}} + \lambda_0 \boldsymbol{\mu}_0}{N+\lambda_0} \right)^\intercal \Big\} \Bigg) \Bigg] + C_2
$$

$$
= -\frac{1}{2} \Bigg[(\nu_N + P + 2) \log |\boldsymbol{\Sigma}| + \operatorname{tr}\left(\boldsymbol{\Sigma}^{-1} \boldsymbol{\Sigma}_N \right) + \lambda_N \left(\boldsymbol{\mu} - \boldsymbol{\mu}_N \right)^\intercal \boldsymbol{\Sigma}^{-1} \left(\boldsymbol{\mu} - \boldsymbol{\mu}_N \right) \Bigg] + C_2.
$$

By identification, we recognise the log-PDF that characterises the Gaussian-inverse-Wishart distribution $\mathcal{NIW}^{-1}(\boldsymbol{\mu}_N, \lambda_N, \boldsymbol{\Sigma}_N, \nu_N)$ with:

- $\boldsymbol{\mu}_N = \dfrac{N\bar{\boldsymbol{y}} + \lambda_0 \boldsymbol{\mu}_0}{N + \lambda_0}$,
- $\lambda_N = \lambda_0 + N$,
- $\boldsymbol{\Sigma}_N = \boldsymbol{\Sigma}_0 + \sum\limits_{n=1}^{N} (\boldsymbol{y}_N - \bar{\boldsymbol{y}})(\boldsymbol{y}_N - \bar{\boldsymbol{y}})^\intercal + \dfrac{\lambda_0 N}{(\lambda_0 + N)} (\bar{\boldsymbol{y}} - \boldsymbol{\mu}_0)(\bar{\boldsymbol{y}} - \boldsymbol{\mu}_0)^\intercal$,
- $\nu_N = \nu_0 + N$.

Once more, we can integrate over $\boldsymbol{\Sigma}$ to compute the mean's marginal posterior distribution by identifying the PDF of the inverse-Wishart distribution $\mathcal{W}^{-1}\Big(\boldsymbol{\Sigma}_N + \lambda_N \left(\boldsymbol{\mu} - \boldsymbol{\mu}_N \right) \left(\boldsymbol{\mu} - \boldsymbol{\mu}_N \right)^\intercal,$ $\nu_N + 1 \Big)$ and by reorganising the terms:

$$
\begin{aligned}
p(\boldsymbol{\mu} \mid \boldsymbol{y}) &= \int p(\boldsymbol{\mu}, \boldsymbol{\Sigma} \mid \boldsymbol{y}) \,\mathrm{d}\boldsymbol{\Sigma} \\
&= \frac{\lambda_N^{\frac{P}{2}} |\boldsymbol{\Sigma}_{\boldsymbol{N}}|^{\frac{\nu_N}{2}}}{(2\pi)^{\frac{P}{2}} 2^{\frac{P\nu_N}{2}} \Gamma_P\left(\frac{\nu_N}{2}\right)} \\
&\quad \times \int |\boldsymbol{\Sigma}|^{-\frac{\nu_N + P + 2}{2}} \exp\left(-\frac{1}{2} \left(\operatorname{tr}\left(\boldsymbol{\Sigma}_N \boldsymbol{\Sigma}^{-1} \right) - \frac{\lambda_N}{2} \left(\boldsymbol{\mu} - \boldsymbol{\mu}_N \right)^\intercal \boldsymbol{\Sigma}^{-1} \left(\boldsymbol{\mu} - \boldsymbol{\mu}_N \right) \right) \right) \mathrm{d}\boldsymbol{\Sigma} \\
&= \frac{\lambda_N^{\frac{P}{2}} |\boldsymbol{\Sigma}_{\boldsymbol{N}}|^{\frac{\nu_N}{2}}}{(2\pi)^{\frac{P}{2}} 2^{\frac{P\nu_N}{2}} \Gamma_P\left(\frac{\nu_N}{2}\right)} \times \frac{2^{\frac{P(\nu_N+1)}{2}} \Gamma_P\left(\frac{\nu_N+1}{2}\right)}{\left|\boldsymbol{\Sigma}_N + \lambda_N \left(\boldsymbol{\mu} - \boldsymbol{\mu}_N \right) \left(\boldsymbol{\mu} - \boldsymbol{\mu}_N \right)^\intercal \right|^{\frac{\nu_N+1}{2}}} \times 1 \\
&= \frac{\pi^{p(p-1)/4} \prod\limits_{p=0}^{P-1} \Gamma\left(\frac{\nu_N + 1 - p}{2}\right)}{\pi^{P(P-1)/4} \prod\limits_{p=1}^{P} \Gamma\left(\frac{\nu_N + 1 - p}{2}\right)} \times \frac{\lambda_N^{\frac{P}{2}}}{\pi^{\frac{P}{2}}} \\
&\quad \times \underbrace{\frac{|\boldsymbol{\Sigma}_N|^{\frac{\nu_N}{2}}}{|\boldsymbol{\Sigma}_N|^{\frac{\nu_N+1}{2}}} \times \left(1 + \lambda_N \left(\boldsymbol{\mu} - \boldsymbol{\mu}_N \right)^\intercal \boldsymbol{\Sigma}_N^{-1} \left(\boldsymbol{\mu} - \boldsymbol{\mu}_N \right) \right)^{-\frac{\nu_N+1}{2}}}_{\text{Matrix determinant lemma}} \\
&= \frac{\Gamma\left(\frac{\nu_N+1}{2}\right)}{\Gamma\left(\frac{\nu_N+1-P}{2}\right)} \times \frac{\left[\lambda_N (\nu_N - P + 1)\right]^{\frac{P}{2}}}{\left[\pi(\nu_N - P + 1)\right]^{\frac{P}{2}} |\boldsymbol{\Sigma}_N|^{\frac{1}{2}}}
\end{aligned}
$$

$$
\times \left(1 + \frac{\lambda_N(\nu_N - P + 1)}{(\nu_N - P + 1)} (\boldsymbol{\mu} - \boldsymbol{\mu}_N)^\intercal \boldsymbol{\Sigma}_N^{-1} (\boldsymbol{\mu} - \boldsymbol{\mu}_N)\right)^{-\frac{\nu_N + 1}{2}}
$$

$$
= \frac{\Gamma\left(\frac{(\nu_N - P + 1) + P}{2}\right)}{\Gamma\left(\frac{\nu_N - P + 1}{2}\right) [\pi(\nu_N - P + 1)]^{\frac{P}{2}} \left|\frac{\boldsymbol{\Sigma}_N}{\lambda_N(\nu_N - P + 1)}\right|^{\frac{1}{2}}}
$$

$$
\times \left(1 + \frac{1}{\nu_N - P + 1} (\boldsymbol{\mu} - \boldsymbol{\mu}_N)^\intercal \left(\frac{\boldsymbol{\Sigma}_N}{\lambda_N(\nu_N - P + 1)}\right)^{-1} (\boldsymbol{\mu} - \boldsymbol{\mu}_N)\right)^{-\frac{(\nu_N - P + 1) + P}{2}}.
$$

The above expression corresponds to the PDF of a multivariate t-distribution $\mathcal{T}_\nu\left(\boldsymbol{\mu}_N, \hat{\boldsymbol{\Sigma}}\right)$, with:

- $\nu = \nu_N - P + 1$,
- $\hat{\boldsymbol{\Sigma}} = \dfrac{\boldsymbol{\Sigma}_N}{\lambda_N(\nu_N - P + 1)}$.

Therefore, we demonstrated that for each group and imputed dataset, the complete-data posterior distribution over $\boldsymbol{\mu}_k$ is a multivariate t-distribution. Thus, following Rubin's rules for multiple imputation (Little and Rubin, 2019) for a small number of imputation draws D, we can propose an approximation to the true posterior distribution (that is only conditioned over observed values):

$$
\begin{aligned}
p\left(\boldsymbol{\mu}_k \mid \boldsymbol{y}_k^{(0)}\right) &= \int p\left(\boldsymbol{\mu}_k \mid \boldsymbol{y}_k^{(0)}, \boldsymbol{y}_k^{(1)}\right) p\left(\boldsymbol{y}_k^{(1)} \mid \boldsymbol{y}_k^{(0)}\right) \mathrm{d}\boldsymbol{y}_k^{(1)} \\
&\simeq \frac{1}{D} \sum_{p=1}^{D} p\left(\boldsymbol{\mu}_k \mid \boldsymbol{y}_k^{(0)}, \tilde{\boldsymbol{y}}_k^{(1),d}\right)
\end{aligned}
$$

Leading to the desired results when evaluating the previously derived posterior distribution on each multiple-imputed dataset. □

C Sensitivity analysis for hyperparameters

In this section, we aim to explore a wide range of prior values for each free hyperparameter (thus excluding prior means, which are always set equal across groups, leading to invariant effects on posterior differences). The tested values in the following experiments are deliberately more extreme than those typically assumed in proteomics studies, to evaluate robustness to strong misspecifications. Sensitivity is measured using two metrics, Negative Log-Likelihood (NLL) and Credible Interval Coverage (CIC_{95}), to quantify both sharpness and uncertainty calibration of the posterior distributions computed with different prior values on the same synthetic datasets. For NLL, the lower the value, the better, whereas CIC_{95} values should be as close as possible to the theoretical threshold 95%.

In all experiments, we generated 1000 synthetic datasets using our simulation scheme with a unique group/condition. Unless otherwise stated, 5 samples are observed for each peptide. The posterior distributions are derived analytically for all the synthetic datasets, using default hyperparameter values, except for the tested one, whose range of values is explicitly stated in the corresponding figures. The true mean of each peptide is used to compute NLL and CIC_{95} metrics, and results are averaged over the 1000 synthetic datasets.

C.1 Sensitivity of α_0, β_0, and λ_0

The sensitivity of hyperparameter priors in univariate ProteoBayes is reported in Figure A. We can observe that both α_0 and λ_0 seem to deteriorate posteriors when set to excessively high values. This result is expected, as λ_0 controls the confidence one places in the prior mean, which can dramatically bias the posterior mean if μ_0 is far from the data. Similarly, α_0 is often thought of as the prior degrees of freedom, and intuitively acts as the number of *pseudo-observations* that may dominate data if set too high. Overall, we can see that all hyperparameters seem robust when maintained at reasonable values for such models, typically below 1, roughly meaning that the prior should not influence the posterior more than one data point would.

C.2 Sensitivity of Σ_0

In Figure B, we displayed the sensitivity of the posteriors to misspecification of the scale matrix Σ_0. To measure different orders of misspecification, we generated data using the Σ_{ref} matrix defined in Section 3.1 *Synthetic datasets*. Then, the off-diagonal terms controlling correlations between peptides are scaled by a factor that we call the *covariance strength*, which varies along the x-axis. We can see that, in most cases, the posteriors are remarkably robust to misspecification of Σ_0, except for very small sample sizes, which slightly deteriorate as we approach a diagonal matrix.

C.3 Sensitivity of ν_0

In Figure C, we evaluated the robustness of ν_0, the prior degrees of freedom. As for its univariate counterpart α_0, we observe that the NLL deteriorates as we increase its value, which justifies our choice to keep it close to the theoretically lowest value, since the minimal constraint is $\nu_0 \geq P - N_K - 1$. Uncertainty remains consistently well-calibrated once we are above the stability threshold described above. We also notice that the effect of ν_0 is reasonably insensitive to the correct specification of Σ_0.

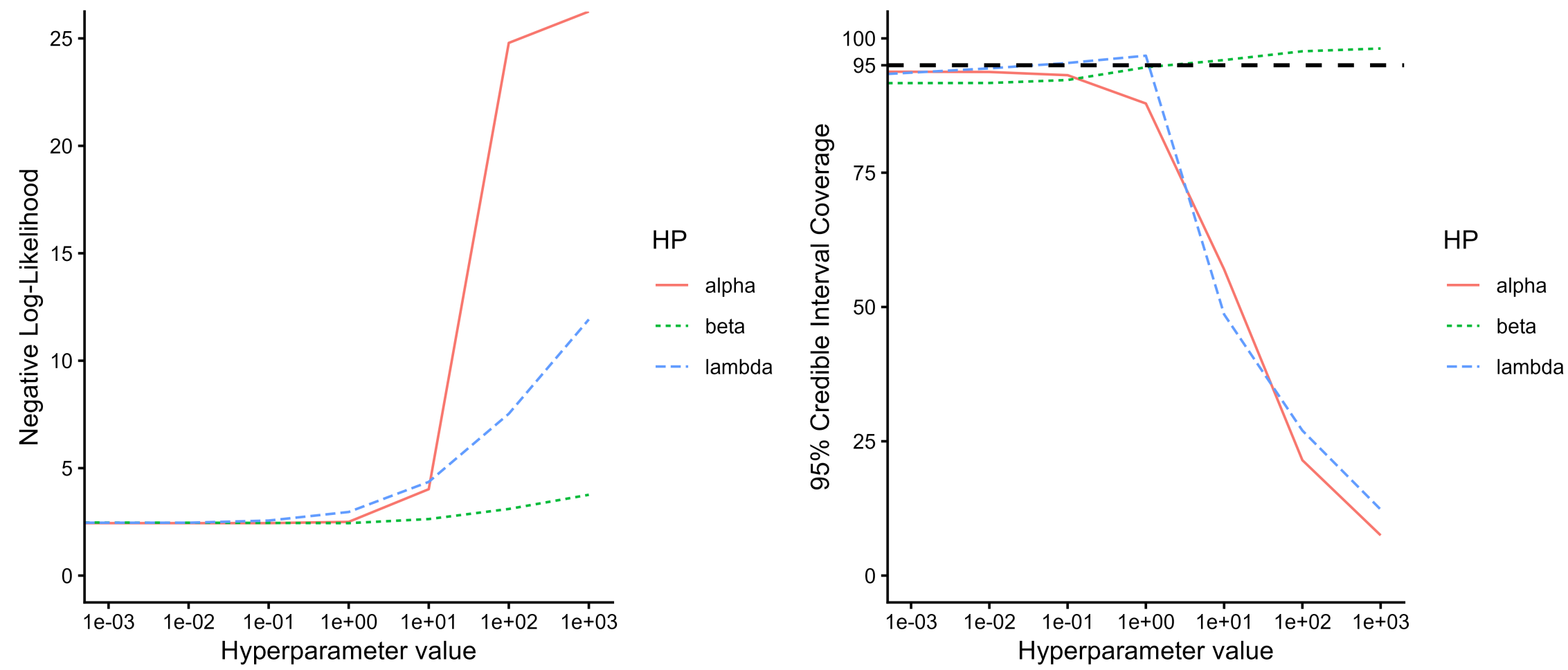

Figure A: Sensitivity analysis for the hyperparameters α_0, β_0, and λ_0 in a univariate ProteoBayes setting. For each curve, one hyperparameter varies along the x-axis while the others are fixed to default values. **Left:** Negative Log-Likelihood of the posterior distribution evaluated on the true mean value. **Right**: 95% Credible Interval Coverage for the true mean value. All results are averaged over 1000 runs of the simulation scheme.

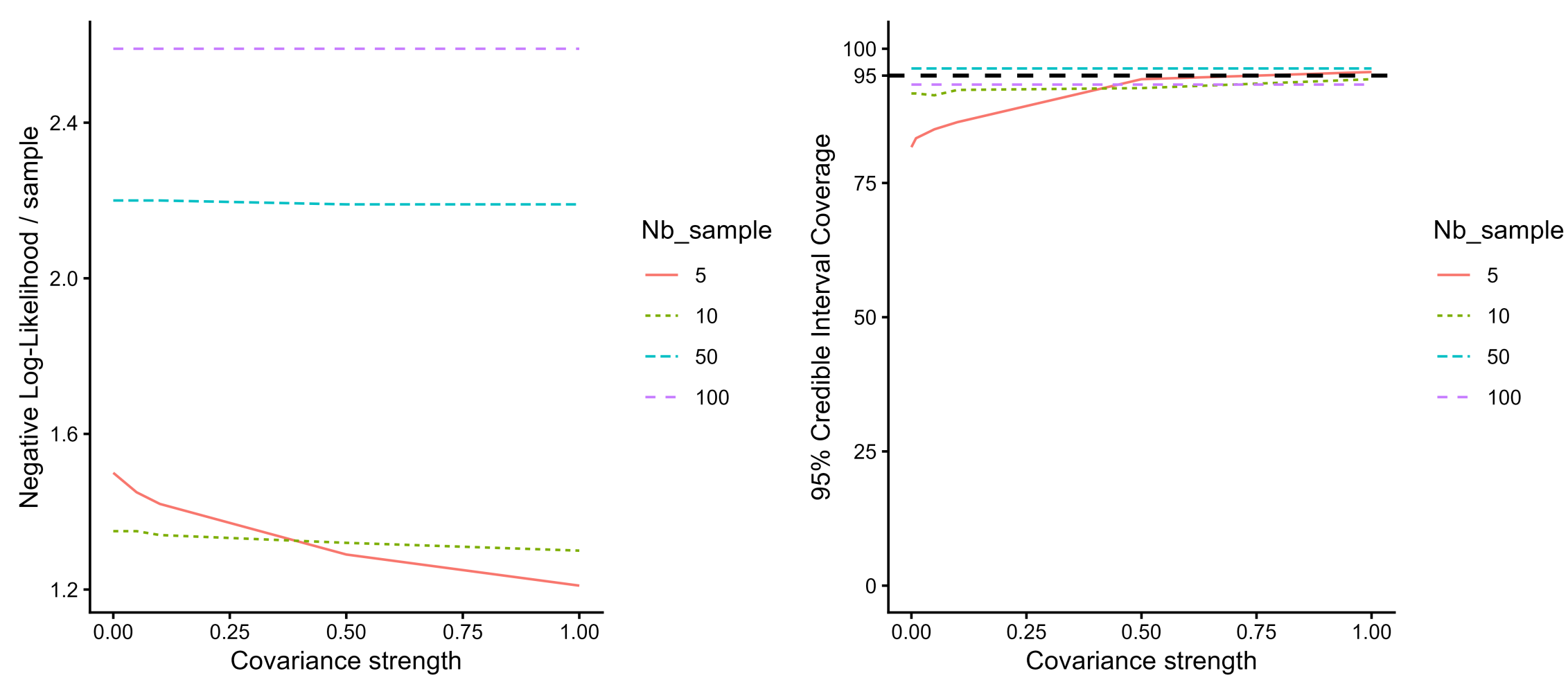

Figure B: Sensitivity analysis for the hyperparameter Σ_0 in a multivariate ProteoBayes setting. For each curve, posterior distributions are computed from datasets with the corresponding sample sizes. The off-diagonal terms of the prior matrix Σ_0 are scaled by a factor that varies along the x-axis. **Left:** Negative Log-Likelihood of the posterior distribution evaluated on the true mean value, divided by the number of samples. **Right**: 95% Credible Interval Coverage for the true mean value. All results are averaged over 1000 runs of the simulation scheme.

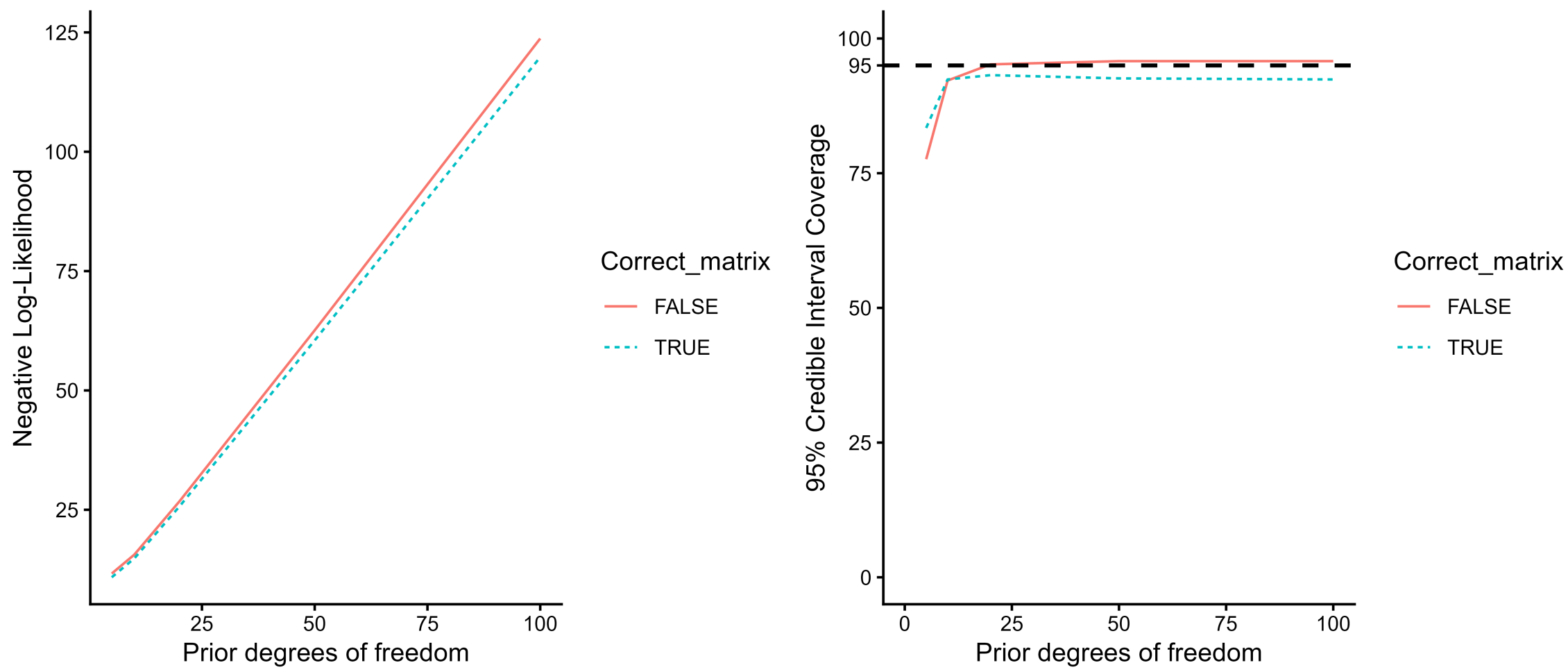

Figure C: Sensitivity analysis for the hyperparameter ν_0 in a multivariate ProteoBayes setting. The prior degrees of freedom vary along the x-axis. The distinct curves correspond to a prior specification of the scale matrix Σ_0 either matching the true covariance structure of the data (TRUE) or an incorrect diagonal matrix (FALSE). **Left:** Negative Log-Likelihood of the posterior distribution evaluated on the true mean value. **Right**: 95% Credible Interval Coverage for the true mean value. All results are averaged over 1000 runs of the simulation scheme.

C.4 Joint effect of α_0 and β_0

In Figure D, we provide an additional insight into the joint effect of the inverse-gamma hyperparameters. This heatmap of errors in uncertainty quantification is computed using wide ranges of prior values for both α_0 and β_0. We observe that low errors are obtained when both are of the same order of magnitude. In particular, although previous Figure A exhibited pathological regimes for high values of α_0, we observe that this behaviour disappears when β_0 increases at the same rate. The values retained as default for the experiments correspond to the $\{\alpha_0 = 0.01, \beta_0 = 0.3\}$ pair, which minimises calibration errors across 1000 simulation runs, although this figure illustrates that many other choices would have been equally valid.

C.5 Uncertainty calibration for different thresholds

To confirm that uncertainty calibration is measured correctly, we depict in Figure E the empirical calibration of the Credible Interval Coverage (CIC) across all probability levels (although 0.95 is used as the default in all experiments). The empirical plain red line overlapping the theoretical dashed line confirms that, for the default set of prior hyperparameters, our credible intervals remains conform to the expected values.

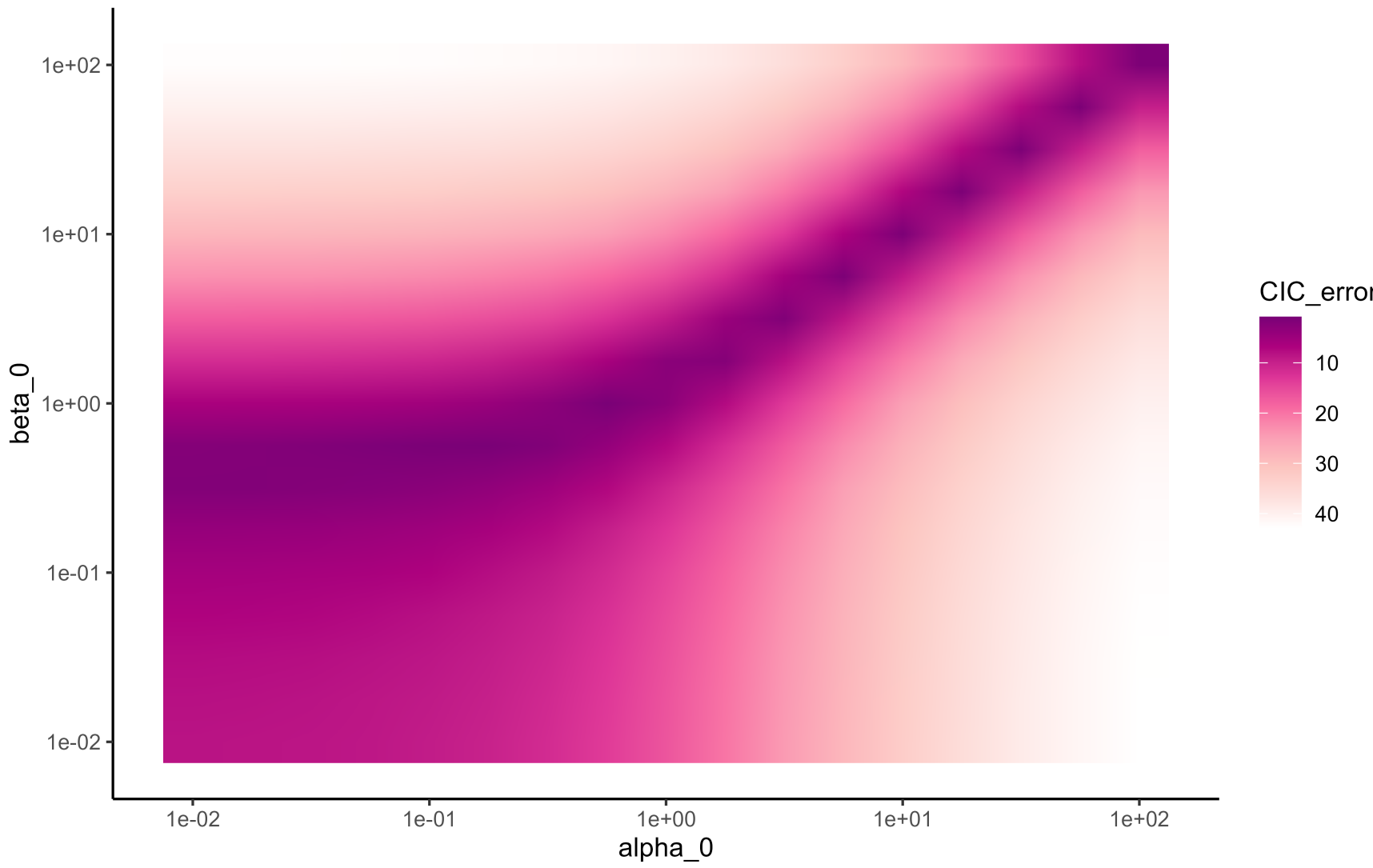

Figure D: Heatmap of errors for the values of the Credible Interval Coverage with respect to α_0 and β_0 values. Empirical errors are computed over 1000 runs on synthetic data according to the simulated scheme with a fixed value of $\lambda_0 = 10^{-10}$.

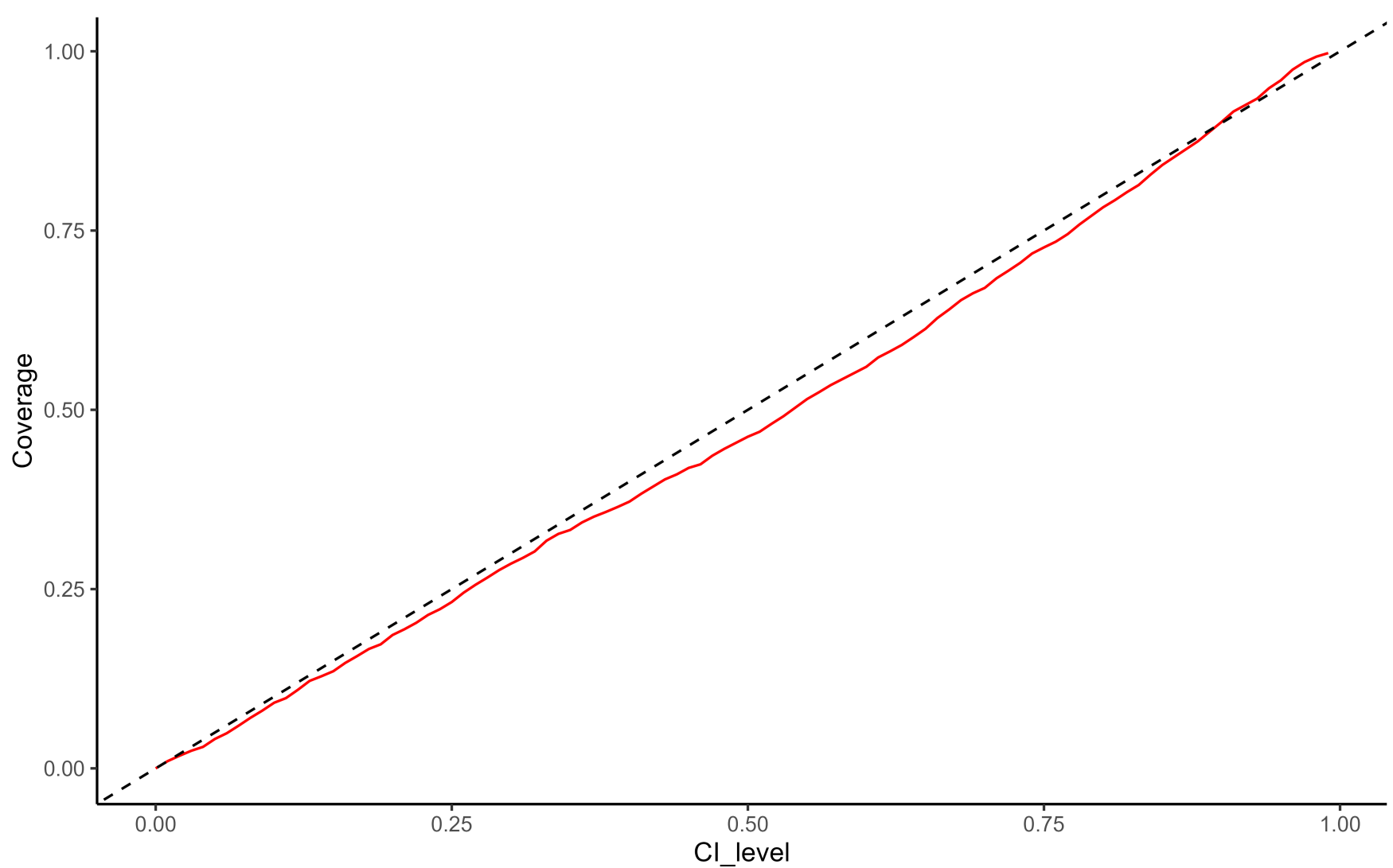

Figure E: Empirical validation of the Credible Interval Coverage (CIC) for all threshold probabilities between 0 and 1. The dashed line represents the theoretical level of the Credible Interval. The red line corresponds to the empirical coverage computed from synthetic data using the simulated scheme.

D Real-data experiments: additional results

Type	Vs. 50 fmol	Nb of peptides	Mean difference			ProteoBayes		
			True	limma	ProteoBayes	CI_{95} width	RMSE	CIC_{95}
UPS	10 amol	101	12.29	5.49 (1.99)	5.49 (1.99)	11.47 (8.07)	5.58 (3.91)	55.45 (49.95)
	50 amol	94	9.97	5.64 (1.96)	5.64 (1.96)	10.52 (8.20)	3.15 (2.68)	55.32 (49.98)
	100 amol	108	8.97	5.88 (1.96)	5.88 (1.96)	10.71 (8.17)	2.19 (2.28)	62.04 (48.76)
	250 amol	181	7.64	5.89 (1.81)	5.89 (1.81)	9.17 (8.04)	1.45 (2.17)	86.19 (34.60)
	500 amol	252	6.64	5.86 (1.29)	5.86 (1.29)	7.95 (7.85)	0.76 (1.20)	99.21 (8.89)
	1 fmol	351	5.64	5.20 (1.06)	5.20 (1.06)	6.20 (7.20)	0.64 (1.08)	92.88 (25.76)
	5 fmol	545	3.32	3.25 (0.56)	3.25 (0.56)	3.62 (5.47)	0.66 (1.11)	88.81 (31.56)
	10 fmol	623	2.32	2.26 (0.56)	2.26 (0.56)	2.79 (4.47)	0.71 (1.34)	88.76 (31.61)
	25 fmol	680	1	0.99 (0.39)	0.99 (0.39)	2.02 (3.25)	0.69 (1.40)	88.38 (32.07)
YEAST	10 amol	19739	0	0.12 (0.41)	0.12 (0.41)	2.89 (4.62)	0.23 (0.53)	99.75 (4.98)
	50 amol	19776	0	0.13 (0.42)	0.13 (0.42)	2.74 (4.41)	0.24 (0.69)	99.69 (5.59)
	100 amol	19749	0	0.14 (0.40)	0.14 (0.40)	2.72 (4.39)	0.22 (0.61)	99.78 (4.66)
	250 amol	19770	0	0.14 (0.42)	0.14 (0.42)	2.76 (4.46)	0.23 (0.64)	99.76 (4.92)
	500 amol	19852	0	0.16 (0.42)	0.16 (0.42)	2.74 (4.40)	0.23 (0.65)	99.83 (4.07)
	1 fmol	19783	0	0.16 (0.41)	0.16 (0.41)	2.72 (4.38)	0.23 (0.57)	99.81 (4.38)
	5 fmol	19768	0	0.14 (0.40)	0.14 (0.40)	2.73 (4.40)	0.23 (0.59)	99.76 (4.92)
	10 fmol	19790	0	0.13 (0.38)	0.13 (0.38)	2.72 (4.40)	0.24 (0.64)	99.66 (5.81)
	25 fmol	19632	0	0.06 (0.35)	0.06 (0.35)	2.83 (4.55)	0.25 (0.64)	99.67 (5.70)

Table B: Results table for the univariate differential analysis of the Bouyssie2020 dataset. All results are averaged over all peptides in each group and reported using the format *Mean (Sd)*.

Type	Vs. 7.54 amol	Nb of peptides	Mean difference			ProteoBayes		
			True	limma	ProteoBayes	CI_{95} width	RMSE	CIC_{95}
UPS	0.75 amol	382	3.33	2.81 (1.77)	2.81 (1.77)	3.34 (4.46)	0.88 (1.62)	91.10 (28.51)
	0.83 amol	382	3.18	2.82 (1.69)	2.82 (1.69)	3.45 (4.75)	0.86 (1.50)	91.62 (27.74)
	1.07 amol	382	2.82	2.56 (1.51)	2.56 (1.51)	3.20 (4.66)	0.71 (1.25)	94.50 (22.82)
	2.04 amol	390	1.89	1.74 (1.34)	1.74 (1.34)	2.63 (3.97)	0.65 (1.04)	93.85 (24.06)
MOUSE	0.75 amol	95599	0	0.03 (0.78)	0.03 (0.78)	1.82 (2.41)	0.46 (1.27)	97.74 (14.86)
	0.83 amol	95591	0	0.02 (0.78)	0.02 (0.78)	1.83 (2.46)	0.45 (1.15)	97.77 (14.76)
	1.07 amol	95588	0	0.02 (0.77)	0.02 (0.77)	1.83 (2.46)	0.45 (1.21)	98.00 (14.00)
	2.04 amol	95553	0	0.01 (0.77)	0.01 (0.77)	1.90 (2.54)	0.46 (1.17)	97.96 (14.14)

Table C: Results table for the univariate differential analysis of the Huang2020 dataset. All results are averaged over all peptides in each group and reported using the format *Mean (Sd)*.

Type	Vs. 10 fmol	Nb of peptides	Mean difference			ProteoBayes		
			True	limma	ProteoBayes	CI_{95} width	RMSE	CIC_{95}
UPS	0.05 fmol	205	7.64	4.21 (2.41)	4.21 (2.41)	7.64 (7.64)	3.20 (3.36)	49.76 (50.12)
	0.25 fmol	350	5.32	4.59 (0.90)	4.59 (0.90)	6.44 (7.12)	0.72 (1.29)	96.29 (18.94)
	0.5 fmol	459	4.32	3.52 (0.87)	3.52 (0.87)	4.71 (5.97)	0.65 (0.89)	94.99 (21.84)
	1.25 fmol	539	3	3.06 (0.72)	3.06 (0.72)	4.82 (5.75)	0.72 (0.99)	91.47 (27.97)
	2.5 fmol	608	2	1.7 (0.49)	1.7 (0.49)	3.35 (4.45)	0.58 (0.92)	92.76 (25.93)
	5 fmol	618	1	1.43 (0.57)	1.43 (0.57)	3.69 (4.78)	0.88 (1.25)	86.89 (33.77)
ARATH	0.05 fmol	15874	0	0.03 (0.60)	0.03 (0.60)	3.25 (4.37)	0.37 (0.77)	99.21 (8.84)
	0.25 fmol	15879	0	0.06 (0.58)	0.06 (0.58)	3.12 (4.25)	0.35 (0.79)	99.31 (8.26)
	0.5 fmol	15989	0	0.07 (0.56)	0.07 (0.56)	3.15 (4.25)	0.33 (0.93)	99.49 (7.10)
	1.25 fmol	16397	0	0.08 (0.61)	0.08 (0.61)	3.74 (4.62)	0.45 (0.90)	98.33 (12.82)
	2.5 fmol	16253	0	0.04 (0.46)	0.04 (0.46)	3.45 (4.61)	0.28 (0.80)	99.73 (5.20)
	5 fmol	16228	0	0.03 (0.51)	0.03 (0.51)	3.88 (4.95)	0.48 (0.88)	98.24 (13.14)

Table D: Results table for the univariate differential analysis of the Chion2022 dataset. All results are averaged over all peptides in each group and reported using the format *Mean (Sd)*.

E Simulation experiment on MNAR data

	MNAR Missing ratio	ProteoBayes					limma p_value	proDA p_value
		Mean difference	RMSE	CIC_{95}	CI_{95} width	TVD		
**Complete data**	0%	1.01 (0.39)	0.32 (0.37	95.00 (21.83)	1.18 (0.26)	0.19 (0.21)	0.08 (0.14)	0.13 (0.18)
**No imputation**	20%	0.98 (0.50)	0.50 (0.56)	77.33 (41.94)	1.07 (0.28)	0.18 (0.22)	0.13 (0.23)	0.12 (0.20)
	50%	0.96 (0.43)	0.80 (0.78)	52.67 (50.01)	1.11 (0.30)	0.21 (0.20)	0.13 (0.23)	0.17 (0.25)
	80%	1.05 (0.56)	1.31 (1.14)	88.28 (32.23)	1.78 (0.45)	0.43 (0.23)	0.15 (0.20)	1.00 (0.00)
**Imputation**	20%	1.08 (0.45)	0.48 (0.51)	67.67 (46.85)	0.85 (0.22)	0.10 (0.17)	0.05 (0.14)	0.07 (0.16)
	50%	1.02 (0.51)	0.82 (0.76)	11.67 (32.16)	0.56 (0.16)	0.06 (0.13)	0.04 (0.12)	0.03 (0.11)
	80%	1.06 (0.65)	1.45 (1.15)	1.67 (12.82)	0.36 (0.07)	0.05 (0.11)	0.03 (0.13)	0.03 (0.12)

Table E: Performance metrics of ProteoBayes, limma, and proDA, for different scenarios of missing data ratios. Observations with the lowest observed values were removed, and imputation is performed by replacing missing values with the average of the observed ones. All results are averaged over 1000 repetitions of the experiments with 10 samples per peptide and reported using the format Mean (Sd).

References

Roderick Little and Donald Rubin. *Statistical Analysis with Missing Data, Third Edition*, volume 26 of *Wiley Series in Probability and Statistics*. Wiley, wiley edition, 2019. ISBN 978-0-470-52679-8.

List of Figures

A Sensitivity analysis for the hyperparameters α_0, β_0, and λ_0 in a univariate ProteoBayes setting. For each curve, one hyperparameter varies along the x-axis while the others are fixed to default values. **Left:** Negative Log-Likelihood of the posterior distribution evaluated on the true mean value. **Right**: 95% Credible Interval Coverage for the true mean value. All results are averaged over 1000 runs of the simulation scheme. . . . 9

B Sensitivity analysis for the hyperparameter Σ_0 in a multivariate ProteoBayes setting. For each curve, posterior distributions are computed from datasets with the corresponding sample sizes. The off-diagonal terms of the prior matrix Σ_0 are scaled by a factor that varies along the x-axis. **Left:** Negative Log-Likelihood of the posterior distribution evaluated on the true mean value, divided by the number of samples. **Right**: 95% Credible Interval Coverage for the true mean value. All results are averaged over 1000 runs of the simulation scheme. . . . 9

C Sensitivity analysis for the hyperparameter ν_0 in a multivariate ProteoBayes setting. The prior degrees of freedom vary along the x-axis. The distinct curves correspond to a prior specification of the scale matrix Σ_0 either matching the true covariance structure of the data (TRUE) or an incorrect diagonal matrix (FALSE). **Left:** Negative Log-Likelihood of the posterior distribution evaluated on the true mean value. **Right**: 95% Credible Interval Coverage for the true mean value. All results are averaged over 1000 runs of the simulation scheme. . . . 10

D Heatmap of errors for the values of the Credible Interval Coverage with respect to α_0 and β_0 values. Empirical errors are computed over 1000 runs on synthetic data according to the simulated scheme with a fixed value of $\lambda_0 = 10^{-10}$. 11
E Empirical validation of the Credible Interval Coverage (CIC) for all threshold probabilities between 0 and 1. The dashed line represents the theoretical level of the Credible Interval. The red line corresponds to the empirical coverage computed from synthetic data using the simulated scheme. 11

List of Tables

A Notation and abbreviation table. 3
B Results table for the univariate differential analysis of the Bouyssie2020 dataset. All results are averaged over all peptides in each group and reported using the format *Mean (Sd)*. 12
C Results table for the univariate differential analysis of the Huang2020 dataset. All results are averaged over all peptides in each group and reported using the format *Mean (Sd)*. 13
D Results table for the univariate differential analysis of the Chion2022 dataset. All results are averaged over all peptides in each group and reported using the format *Mean (Sd)*. 13
E Performance metrics of ProteoBayes, limma, and proDA, for different scenarios of missing data ratios. Observations with the lowest observed values were removed, and imputation is performed by replacing missing values with the average of the observed ones. All results are averaged over 1000 repetitions of the experiments with 10 samples per peptide and reported using the format Mean (Sd). 14
