# Supplementary material for: A Bayesian framework for multivariate differential analysis
Source: PLoS Comput Biol. 2026 Sep 2;22(9):e1014637. doi: 10.1371/journal.pcbi.1014637 (PMC13561429; doi:10.1371/journal.pcbi.1014637)
Supplement: Supplementary file 2 [file pcbi.1014637.s002.pdf]

Recommendation: Major Revision

Summary of the manuscript

The authors present ProteoBayes, a fully Bayesian framework for differential analysis in quantitative proteomics that achieves closed-form posterior inference through conjugate Normal-Inverse-Gamma (univariate) and Normal-Inverse-Wishart (multivariate) priors. The method provides well-calibrated uncertainty quantification at computational costs comparable to frequentist moderated t-tests while naturally handling missing data and inter-analyte correlations. Validated on synthetic datasets and four real spike-in studies, ProteoBayes offers richer probabilistic outputs (overlap coefficients, posterior probabilities) as alternatives to p-values. The work represents a philosophically and practically appealing advance, though it would benefit from expanded prior sensitivity analysis, scalability considerations for high-dimensional settings, and broader benchmarking against recent Bayesian competitors.

Major strengths

The closed-form analytical derivations are elegant and rigorously presented, making the method computationally competitive with frequentist moderated t-tests despite being fully Bayesian.

The multivariate extension that jointly models correlated analytes (e.g., peptides from the same protein) addresses a genuine gap in standard proteomics pipelines.

Treatment of missing data is thoughtful: the recommendation to ignore missing values in univariate settings (under MAR) is well-justified theoretically and empirically, and the proposed multivariate imputation approximation is pragmatic.

Emphasis on calibrated uncertainty and interpretable probabilistic statements (overlap coefficients, posterior probabilities) is timely and valuable for practical decision-making in biology.

Open-source implementation (R package on CRAN/GitHub with Shiny app) and thorough benchmarking enhance reproducibility and usability.

Major comments

Prior sensitivity: The default priors are intentionally vague (e.g., lambda_0 = 10^-10 effectively removes prior mean influence). While supplementary figures explore some sensitivity, a more systematic analysis across realistic proteomics scenarios would strengthen claims of robustness. Consider adding results for moderately informative priors on variance (e.g., reflecting typical peptide variance ranges).

Scalability in high-dimensional multivariate settings: The method is demonstrated up to ~1,000 correlated analytes, but proteomics datasets can involve thousands of peptides per protein group. The authors acknowledge the curse of dimensionality - could they discuss or briefly evaluate structured covariance approximations (e.g., diagonal + low-rank, factor models) for larger groups?

Missing not at random (MNAR): The manuscript appropriately limits scope to MAR, but many proteomics

missingness mechanisms are MNAR (e.g., below detection limit). A short discussion of limitations and potential extensions (e.g., selection/Heckman models) would be helpful.

Benchmark breadth: Comparisons focus primarily on limma and basic t-tests. Including recent Bayesian or probabilistic methods (e.g., proDA, Triqler, IsoBayes, MSqRob) would better contextualize novelty and performance, especially for protein-level inference.

Minor comments

The overlap coefficient is an interesting alternative to traditional criteria. A brief comparison to region of practical equivalence (ROPE) approaches in the Bayesian decision-making literature could enrich the discussion.

Protein-level aggregation using N+ (sum of posterior probabilities across peptides) is clever but heuristic - additional validation on datasets with known isoform complexity would be reassuring.

Clarify whether the multivariate model assumes equal variances across conditions (as in limma) or allows condition-specific covariances.

The paper claims "similar computational cost as hypothesis testing" but provides limited empirical timing comparisons. A table showing wall-clock runtime for ProteoBayes vs. limma/MSstats across varying dataset sizes (e.g., 100, 1,000, 10,000 peptides) would substantiate this claim.

Overall assessment

This is a strong, technically sound contribution that offers a practical and philosophically appealing Bayesian alternative to current proteomics differential analysis standards. With moderate expansion on prior robustness, scalability considerations, benchmark scope, and runtime evidence, it would be suitable for publication in PLOS Computational Biology.

Confidential comments to editor

No conflicts of interest. The work is clearly within the journal's scope as a methods advance in computational biology with direct relevance to proteomics. The analytical derivations and empirical validation appear solid. I recommend Major Revision to address the points above; I would be happy to re-review if needed.
