# Supplementary material for: A Bayesian framework for multivariate differential analysis
Source: PLoS Comput Biol. 2026 Sep 2;22(9):e1014637. doi: 10.1371/journal.pcbi.1014637 (PMC13561429; doi:10.1371/journal.pcbi.1014637)
Supplement: Supplementary file 3 [file pcbi.1014637.s004.pdf]

Dear editors,

Thank you for giving us the opportunity to submit a revised draft of the manuscript "A Bayesian Framework for Multivariate Differential Analysis" for publication in *PLOS Computational Biology*. We appreciate the time and effort you and the reviewers dedicated to providing feedback on our manuscript, and we are grateful for the insightful comments. Changes in the manuscript were highlighted in blue in the tracked version and in our responses below. As several suggestions were shared by different reviewers, we may kindly refer to previous answers with the format **Comment X.X** when appropriate.

Reviewer 1

1.1. The prior assumptions adopted in the multivariate model remain relatively strong, and the range of applicability of this model would benefit from further discussion.

We agree that the multivariate formulation requires a clearer discussion of its range of applicability. Strictly speaking, the multivariate model does not rely on assumptions of a different nature than the univariate one: it is the natural multivariate extension of the same conjugate Gaussian framework, replacing the univariate variance by a covariance matrix and the normal-inverse-gamma prior by its multivariate analogue. However, we agree that this extension is more demanding in practice, because estimating covariance structures from limited replication is substantially harder than estimating independent variances.

We have therefore revised the manuscript to clarify that the multivariate framework is primarily intended for moderate-dimensional groups of correlated analytes, such as peptides within a protein, where covariance modelling is biologically meaningful and statistically feasible, rather than for arbitrarily large high-dimensional blocks. We added in section 2.2 the following paragraph: The multivariate formulation should be viewed as the natural extension of the univariate conjugate Gaussian model to vector-valued observations. Its assumptions are therefore analogous in nature, but the model is more demanding in practice because it requires estimation of covariance structures, which is substantially harder than estimating independent variances from limited replication. Accordingly, we primarily intend this framework for moderate-dimensional groups of correlated analytes, such as peptides within a protein, where explicit covariance modelling is both biologically meaningful and statistically feasible.

1.2. The conclusion that missing values can be ignored relies on the Missing At Random (MAR) assumption, whose practical validity should be emphasized more clearly. I encourage the authors to explicitly state that the proposed approach may fail under Missing Not At Random (MNAR) mechanisms, and to discuss its relationship and differences with existing MNAR-oriented methods, such as Triqler or selection models.

We agree that we had to make the practical validity of the MAR assumption more explicit. Therefore, we made the following changes.

- In the abstract, we explicitly mentioned the MAR assumption.
  "We also demonstrate that, in this Bayesian treatment, missing at random data should generally be ignored in univariate settings, and further derive a tailored approximation that handles multiple imputation for the multivariate setting."

- In section 2.3, we included the limits in MNAR settings and referred to Heckman-type models and Triqler (as also suggested by Reviewer 2).
  We replaced this text: "The more complicated Missing Not At Random (MNAR) scenario remains to be studied and is outside the scope of the present paper. However, in differential proteomics, the most common practice is to analyse each peptide independently, under the MAR assumption (as MNAR observations are generally filtered out at preprocessing). Under these assumptions, the Bayesian framework addresses the missing-data issue in a natural and somewhat more straightforward way." by "Under MAR, the missingness mechanism is ignorable for inference on the means, and the observed-data posterior is sufficient. This does not extend to Missing Not At Random (MNAR) settings, where missingness depends on the unobserved abundance itself, for instance, through censoring below a detection limit. In such cases, missingness is informative and should be modelled explicitly, as in selection or Heckman-type models (Heckman, 1979; Orwoll et al., 2020) and related probabilistic approaches such as proDA (Ahlmann-Eltze and Anders, 2019) or Triqler (The and Käll, 2021). Therefore, our result should be interpreted as a no-imputation result for the univariate MAR setting, not as a general recommendation for all proteomics missing-data problems.
- In section 3.7.3, we changed the title to "The mirage of MAR imputed data" and clarified our statement about the validity of imputation methods for proteomics data.
  We replaced this text: "Imputation does not actually make sense in one dimension since, by definition, a missing data point is simply equivalent to an unobserved one, as we shall obtain more information only by collecting more data. Therefore, one should be careful when dealing with imputed datasets and keep in mind that imputation *creates* new data points that do not bear any additional information. Thus, there is a risk of artificially reducing the uncertainty of our estimated posterior distributions by including more data points in the computations than were genuinely observed." by "Our claim is not that imputation is always inappropriate in proteomics, nor that all missingness mechanisms are ignorable. Rather, in the univariate MAR setting considered here, replacing missing values with artificial observations can create the illusion of increased information, thereby distorting uncertainty quantification."
- We also specified the MAR assumption in the conclusion. We replaced the following text: "We recalled that missing data should be ignored in an univariate setting (provided they occur at random) to preserve the accuracy of uncertainty quantification in statistical analyses." by "We showed that, in the univariate setting with independent peptides and under MAR assumption, missing values can be integrated out analytically, so that imputation is unnecessary and may even degrade uncertainty quantification."
- To provide additional insights and strengthen empirical evaluation, we also extended the missing data experiments reported in Table 5. The naive t-tests were replaced with proDA results. The RMSE and Total Variation Distance (TVD) metrics were also added to better assess the validity of ProteoBayes across various scenarios. We further extended this evaluation, in Table S5.5 of the Supplementary, with an experiment that emulates an MNAR context to test the robustness of the different methods to a large range of MNAR proportions. Those results were discussed in the following paragraph: "Even the *proDA* algorithm, specifically designed to deal with missing data and avoid imputation, struggles to provide meaningful results in our experiments, and often failed to converge in really sparse regimes (as highlighted by pathological results for 80% missing data). While our claim about ignoring missing data holds only under MAR hypotheses, we conducted additional experiments to evaluate empirical robust-

ness to MNAR. In Table S5.5, instead of randomly sampling missing data, we systematically removed the lowest observed values (to emulate the detection threshold often encountered in proteomics), while still increasing the missingness ratio in both imputation and no-imputation contexts. We can observe that calibration metrics deteriorate, as expected, with increased RMSE and damaged credible intervals. However, this behaviour seems much worse when imputing missing data, leading to strong overconfidence. In terms of mean difference and total variation distance, ProteoBayes remains reasonably robust and cautious, whereas both limma and proDA seem to be consistently overconfident."

1.3. The authors propose probabilistic inference criteria such as the overlap coefficient, which is conceptually appealing. However, from a practical user's perspective, determining how large a probability is sufficient to declare a difference still requires subjective judgment. Therefore, it would be helpful to provide clearer usage guidelines, for example, recommended thresholds or an approximate correspondence with commonly used FDR-based criteria.

We thank the reviewer for highlighting the need for clearer practical guidance. We agree that although posterior probabilities and overlap-based summaries are more interpretable than p-values, practitioners still need an operational reporting rule. We therefore revised the manuscript to make this point more explicit. In the univariate setting, we now recommend interpreting posterior evidence in conjunction with a minimum effect size of practical relevance, rather than relying solely on the probability that the effect is non-zero. More specifically, we propose a pragmatic default rule that requires at least 95% posterior probability that the effect exceeds a biologically meaningful threshold in one direction. In the multivariate setting, we similarly clarify that the posterior distribution of N^+ should be summarised through probabilities of the form $\Pr(N^+ \geq k \mid \text{data})$ or $\Pr(N^+ \leq D - k \mid \text{data})$, where k reflects the minimum number of concordant peptides regarded as meaningful. We also clarify that these thresholds should be viewed as practical reporting conventions rather than universal decision rules or formal FDR guarantees.

Hence, we added at the end of section 3.8.1 the following text: "In practice, we recommend summarising the posterior of N^+ through probabilities of the form $Pr(N^+ \geq k \mid \text{data})$ or $Pr(N^+ \leq D - k \mid \text{data})$, where k encodes the minimum number of concordant peptides regarded as biologically meaningful. Large values provide strong evidence for a directional protein-level difference. In this sense, N^+ should be viewed as a continuous measure of evidence. The appropriate reporting threshold depends on the study objective, with confirmatory analyses generally requiring stronger evidence than exploratory ones.". To remain consistent with our claims to favour probabilistic measurements for decision making, we now introduce more formally the overlap coefficient (and the equivalent Total Variation Distance) in a dedicated Section 3.4 The overlap coefficient and the total variation distance. Those two quantities are illustrated in a new Figure 3, which should help the intuition of readers less familiar with probabilistic inference.

1.4. Some notation becomes dense when switching between univariate and multivariate settings (e.g. α, ν, λ). Including a notation table would improve readability.

We included a table of notations and abbreviations in the Supplementary Information.

1.5. Algorithms 1 and 2 could benefit from an additional intuitive explanation to facilitate understanding by readers without a strong Bayesian background

We agree that the pseudocode alone may appear somewhat abstract to readers less familiar with Bayesian inference. We therefore added a short intuitive explanation around Algorithms 1 and 2 to explain the underlying logic in plain language. In particular, we now clarify that the procedure with: "The algorithm first updates the parameters of the posterior distribution of the group means from the observed (and possibly imputed) data. Then, an imputation index is randomly chosen (optional), and samples of the group means are repeatedly drawn from the associated posteriors. Finally, the sampled means are compared across conditions to obtain an empirical posterior distribution of the mean difference." We believe this additional explanation makes the algorithms more accessible without changing their formal content.

1.6. Certain sections (e.g., Section 3.6.2) could be moderately shortened by reducing repetitive descriptions, allowing more space for a discussion of the limitations of the proposed method.

We agree with the reviewer that some paragraphs, particularly in Section 3.6.2 could be lengthy or redundant. We tried our best to remove or shorten repetitive sentences throughout the paper to accommodate the suggested modifications and additional results.

Reviewer 2

2.1. Prior sensitivity: The default priors are intentionally vague (e.g., $\lambda_0 = 10^{-}10$ effectively removes prior mean influence). While supplementary figures explore some sensitivity, a more systematic analysis across realistic proteomics scenarios would strengthen claims of robustness. Consider adding results for moderately informative priors on variance (e.g., reflecting typical peptide variance ranges).

We agree that the hyperparameter sensitivity and robustness were not sufficiently assessed in the original version. This topic is now extensively discussed in a dedicated section of the Supplementary (Section 2, *Sensitivity analysis for hyperparameters*), which is referenced in Section 3.5, *Choice of hyperparameters*, of the main text. We explored a wide range of prior values for each free hyperparameter (thus excluding prior means, which are always set equal across groups, leading to invariant effects on posterior differences). The tested values are deliberately more extreme than those typically assumed in proteomics studies, to evaluate robustness to strong misspecifications. Sensitivity is measured using two metrics, Negative Log-Likelihood (NLL) and Credible Interval Coverage (CIC_{95}), to quantify both the sharpness and the uncertainty calibration of the posterior distributions computed with different prior values on the same synthetic datasets.

2.2. Scalability in high-dimensional multivariate settings: The method is demonstrated up to $\sim 1,000$ correlated analytes, but proteomics datasets can involve thousands of peptides per protein group. The authors acknowledge the curse of dimensionality - could they discuss or briefly evaluate structured covariance approximations (e.g., diagonal + low-rank, factor models) for larger groups?.

As shown in Table 2, which reports runtimes, the multivariate algorithm remains usable for 10,000 correlated peptides at a reasonable (though higher) cost. That being said, it is more than likely that estimating such high-dimensional covariance matrices, particularly from a small number of samples, may yield poor results. We would strongly advise assuming a block-diagonal structure for the whole dataset in such contexts, where each sub-block could contain a few dozen to hundreds of correlated peptides (see also **Comment 1.1** that describes a related paragraph added to the main

text). Such an assumption is immediately compatible with the current version of the model and the associated implementation.

The reason we invoked the curse of dimensionality is not directly related to scalability. Even with a reasonable number of peptides (let's say 10 or 20), we already face severe problems in comparing multivariate probability distributions in a meaningful way, as uncertainty quantification across all possible permutations grows exponentially with the number of peptides, and so does the distance between distributions in high dimensions. This is why we came up with N_+ as a measure of differential status. We argue that this proposal is probably one of the only meaningful quantities that one can compute for inference in multivariate differential analysis contexts.

2.3. Missing not at random (MNAR): The manuscript appropriately limits scope to MAR, but many proteomics missingness mechanisms are MNAR (e.g., below detection limit). A short discussion of limitations and potential extensions (e.g., selection/Heckman models) would be helpful.

We thank the reviewer for raising this point. We agree that the no-imputation result must be interpreted within the MAR setting only, and that many proteomics missingness mechanisms may instead be MNAR. In response, we revised the manuscript to make this limitation much more explicit. In section 2.3, we included the limits in MNAR settings and referred to Heckman-type models and Triqler:
We replaced this text: "The more complicated Missing Not At Random (MNAR) scenario remains to be studied and is outside the scope of the present paper. However, in differential proteomics, the most common practice is to analyse each peptide independently, under the MAR assumption (as MNAR observations are generally filtered out at preprocessing). Under these assumptions, the Bayesian framework addresses the missing-data issue in a natural and somewhat more straightforward way." by "Under MAR, the missingness mechanism is ignorable for inference on the means, and the observed-data posterior is sufficient. This does not extend to Missing Not At Random (MNAR) settings, where missingness depends on the unobserved abundance itself, for instance through censoring below a detection limit. In such cases, missingness is informative and should be modelled explicitly, as in selection or Heckman-type models (Heckman, 1979; Orwoll et al., 2020) and related probabilistic approaches such as Triqler (The and Käll, 2021). Therefore, our result should be interpreted as a no-imputation result for the univariate MAR setting, not as a general recommendation for all proteomics missing-data problems. See also **Comment 1.2** for additional details.

2.4. Benchmark breadth: Comparisons focus primarily on limma and basic t-tests. Including recent Bayesian or probabilistic methods (e.g., proDA, Triqler, IsoBayes, MSqRob) would better contextualize novelty and performance, especially for protein-level inference.

We thank the reviewer for pointing out those methods, which are indeed more legitimate competitors, as the original study was too narrow. We therefore included proDA (appropriate for missing-data experiments) and MSqRob (elsewhere) in our empirical comparisons. Unfortunately, we were unable to evaluate Triqler or IsoBayes as baselines because they operate at different levels of the proteomics inference pipeline. Our method focuses on (multivariate) modelling (correlated) peptide intensities, whereas Triqler propagates identification uncertainty and IsoBayes targets isobaric quantification. From a practical point of view, Triqler is also built as a complete pipeline, from

which we could not extract the differential analysis module. Finally, IsoBayes requires additional information on isoforms that we simply did not observe in our datasets, and could not adequately simulate within our modelling scheme.

Therefore, the mention of these two new methods was added at the beginning of the Experiments section: We also compared our method to proDA (Ahlmann-Eltze and Anders, 2020), which constructs a probabilistic dropout model to avoid imputing missing values, and MSqRob (Goeminne et al., 2018), a robust regression framework that performs peptide-level inference with empirical variance stabilisation.

Tables were updated accordingly, and comments were revised to reflect the results of those new methods.

2.5. The overlap coefficient is an interesting alternative to traditional criteria. A brief comparison to region of practical equivalence (ROPE) approaches in the Bayesian decision-making literature could enrich the discussion.

We agree that the relation to ROPE-style Bayesian decision rules is worth clarifying. In the revised manuscript, we now explain that our perspective is close in spirit to ROPE in that it emphasises practical relevance and effect size, rather than mere departure from zero. However, our goal is not to introduce a formal Bayesian hypothesis-testing procedure or a strict accept/reject rule. Instead, we introduced in the newly added section *3.4 The overlap coefficient and the total variation distance*, the OVL and the TVD primarily as continuous summaries of posterior separation, which are intended to inform interpretation rather than replace it with a binary decision. We have therefore revised the text to state more clearly that any reporting threshold should be chosen in light of the scientific objective and a practically relevant effect size, and that the proposed quantities are reported as continuous measures of evidence rather than formal testing criteria: "In practice, it should be interpreted more as a continuous measure of evidence rather than as a universal binary decision rule. Any reporting threshold should therefore be chosen in light of the scientific objective and a practically relevant effect size, in a way that is conceptually related to ROPE-style decision rules (Krusche, 2018), although we do not use it here as a strict binary testing device. For clarity, we chose to report TVD in the following experiments, as it aligns more closely with the traditional *rejection* viewpoint of hypothesis testing and is likely more straightforward for readers less familiar with the Bayesian paradigm."

2.6. Protein-level aggregation using N+ (sum of posterior probabilities across peptides) is clever but heuristic - additional validation on datasets with known isoform complexity would be reassuring.

We agree that N^+ should be interpreted as a pragmatic summary of coordinated peptide-level directionality within a protein group, rather than as an explicit model of isoform structure. Because it is invariant to peptide permutations, it captures how many peptides support a directional shift, but not which peptides do, and therefore does not explicitly represent isoform-specific peptide subsets or shared-peptide ambiguity. We have clarified this point in the revised manuscript in section 3.8.1: "We also stress that N^+ is a pragmatic summary of coordinated peptide-level directionality within a protein group. Because it is invariant to peptide permutations, it does not explicitly model isoform-specific peptide subsets or ambiguous peptide-to-isoform assignments."

2.7. Clarify whether the multivariate model assumes equal variances across conditions

(as in limma) or allows condition-specific covariances.

In ProteoBayes, the multivariate model allows condition-specific covariance matrices: each group (biological condition) has its own covariance parameter Σ_k, and inference is performed separately for each group before comparing posterior distributions of the corresponding means. What is shared across groups is the prior specification, which we keep identical to ensure an unbiased comparison, not the posterior covariance itself. We have revised the manuscript to make this distinction explicit in section 2.2: "This sharing applies only to the prior specification; the covariance matrices themselves remain group-specific through Σ_k and are updated separately from the observed data in each condition".

2.8. The paper claims "similar computational cost as hypothesis testing" but provides limited empirical timing comparisons. A table showing wall-clock runtime for ProteoBayes vs. limma/MSstats across varying dataset sizes (e.g., 100, 1,000, 10,000 peptides) would substantiate this claim.

We agree that computational cost should be empirically measured, as originally reported in Table 2 in Section *3.7.1 Running time comparison.* Since we added new competing methods following your suggestions, the table has been extended to include the corresponding runtimes for proDA and MSqRob from 10 to 10,000 peptides.

Reviewer 3

3.1. The paper recommends to ignore missingness in the case of MAR, however other types of missingness is out of scope for the paper. It would however be interesting to see the robustness of the method in such scenarios through simulation studies.

Following this suggestion, we conducted an additional experiment that emulates an MNAR context to test the robustness of the different methods to a large range of missing proportions. Those results are reported in Table 1 of the Supplementary. **Comments 1.2 and 2.3** provide more details on all changes made regarding MAR/MNAR data.

3.2. It would be interesting to a senstivity analysis on the choice of prior for the method and how robust in the method to different choices of prior.

We thank all the reviewers for this suggestion, which was indeed lacking in the initial version of the paper. The detailed discussion on this topic can be found under **Comment 2.1** of the present letter.

3.3. In the multivariate simulation study, it would be interesting to see how well the method performs when the sample size is small as computing the covariance matrix would be difficult.

The multivariate simulation study (reported in Table 6) was originally replicated in two sample-size scenarios, with 5 and 100 observed samples per condition, respectively. We believe that 5 observations are representative of the low sample sizes often encountered in proteomics, although we would be happy to reproduce additional experiments if the reviewer suggests a specific range of sample sizes. In this context, we demonstrated that both effect sizes and uncertainty calibration of the multivariate algorithm remain valid and are more appropriate than those of the univariate ones when analytes are correlated.
