# Supplementary material for: A Bayesian framework for multivariate differential analysis
Source: PLoS Comput Biol. 2026 Sep 2;22(9):e1014637. doi: 10.1371/journal.pcbi.1014637 (PMC13561429; doi:10.1371/journal.pcbi.1014637)
Supplement: Peer review history

# Peer review: Decision Letter 0

**DOI:** 10.1371/journal.pcbi.1014637.r001

**Authors:** Ilya Ioshikhes, Lun Hu

4 Feb 2026

A Bayesian Framework for Multivariate Differential Analysis

PLOS Computational Biology

Dear Dr. Chion,

Thank you for submitting your manuscript to PLOS Computational Biology. After careful consideration, we feel that it has merit but does not fully meet PLOS Computational Biology's publication criteria as it currently stands. Therefore, we invite you to submit a revised version of the manuscript that addresses the points raised during the review process.

Please submit your revised manuscript by Apr 06 2026 11:59PM. If you will need more time than this to complete your revisions, please reply to this message or contact the journal office at ploscompbiol@plos.org. When you're ready to submit your revision, log on to https://www.editorialmanager.com/pcompbiol/ and select the 'Submissions Needing Revision' folder to locate your manuscript file.

Please include the following items when submitting your revised manuscript:

* A letter that responds to each point raised by the editor and reviewer(s). You should upload this letter as a separate file labeled 'Response to Reviewers'. This file does not need to include responses to formatting updates and technical items listed in the 'Journal Requirements' section below.

* A marked-up copy of your manuscript that highlights changes made to the original version. You should upload this as a separate file labeled 'Revised Manuscript with Track Changes'.

* An unmarked version of your revised paper without tracked changes. You should upload this as a separate file labeled 'Manuscript'.

If you would like to make changes to your financial disclosure, competing interests statement, or data availability statement, please make these updates within the submission form at the time of resubmission. Guidelines for resubmitting your figure files are available below the reviewer comments at the end of this letter

We look forward to receiving your revised manuscript.

Kind regards,

Lun Hu

Academic Editor

PLOS Computational Biology

Ilya Ioshikhes

Section Editor

PLOS Computational Biology

**Additional Editor Comments:**

I received three review reports, and all reviewers found merit in this work. However, they raised several critical issues, such as robustness evaluation for the missingness case, the discussion on the recommended threshold of large probability, and comparison with SOTA baselines. Due to these concerns, authors should carefully revise their manuscript for a major revision.

**Journal Requirements:**

1) We ask that a manuscript source file is provided at Revision. Please upload your manuscript file as a .doc, .docx, .rtf or .tex. If you are providing a .tex file, please upload it under the item type u2018LaTeX Source Fileu2019 and leave your .pdf version as the item type u2018Manuscriptu2019.

2) Please provide an Author Summary. This should appear in your manuscript between the Abstract (if applicable) and the Introduction, and should be 150-200 words long. The aim should be to make your findings accessible to a wide audience that includes both scientists and non-scientists. Sample summaries can be found on our website under Submission Guidelines:

https://journals.plos.org/ploscompbiol/s/submission-guidelines#loc-parts-of-a-submission

3) Please upload all main figures as separate Figure files in .tif or .eps format. For more information about how to convert and format your figure files please see our guidelines:

https://journals.plos.org/ploscompbiol/s/figures

4) We notice that your supplementary Figures, and Tables are included in the manuscript file. Please remove them and upload them with the file type 'Supporting Information'. Please ensure that each Supporting Information file has a legend listed in the manuscript after the references list.

5) Please amend your detailed Financial Disclosure statement. This is published with the article. It must therefore be completed in full sentences and contain the exact wording you wish to be published.

- State the initials, alongside each funding source, of each author to receive each grant. For example: "This work was supported by the National Institutes of Health (####### to AM; ###### to CJ) and the National Science Foundation (###### to AM)."

- State what role the funders took in the study. If the funders had no role in your study, please state: "The funders had no role in study design, data collection and analysis, decision to publish, or preparation of the manuscript.".

If you did not receive any funding for this study, please simply state: u201cThe authors received no specific funding for this work.u201d

**Reviewers' comments:**

Reviewer's Responses to Questions

**Comments to the Authors:**

**Please note here if the review is uploaded as an attachment.**

Reviewer #1: The manuscript presents a fully Bayesian framework for univariate and multivariate differential analysis that provides calibrated uncertainty quantification, naturally handles missing data and inter-element correlations, and offers intuitive probabilistic inference criteria as a principled and computationally efficient alternative to standard hypothesis-testing approaches.

The authors have made substantial efforts in developing the multivariate differential analysis framework; however, my main concerns are as follows:

1.The prior assumptions adopted in the multivariate model remain relatively strong, and the range of applicability of this model would benefit from further discussion.

2.The conclusion that missing values can be ignored relies on the Missing At Random (MAR) assumption, whose practical validity should be emphasized more clearly. I encourage the authors to explicitly state that the proposed approach may fail under Missing Not At Random (MNAR) mechanisms, and to discuss its relationship and differences with existing MNAR-oriented methods, such as Triqler or selection models.

3.The authors propose probabilistic inference criteria such as the overlap coefficient, which is conceptually appealing. However, from a practical user’s perspective, determining how large a probability is sufficient to declare a difference still requires subjective judgment. Therefore, it would be helpful to provide clearer usage guidelines, for example recommended thresholds or an approximate correspondence with commonly used FDR-based criteria.

4.Some notation becomes dense when switching between univariate and multivariate settings (e.g., α, ν, λ). Including a notation table would improve readability.

5.Algorithms 1 and 2 could benefit from an additional intuitive explanation to facilitate understanding by readers without a strong Bayesian background.

6.Certain sections (e.g., Section 3.6.2) could be moderately shortened by reducing repetitive descriptions, allowing more space for a discussion of the limitations of the proposed method.

Reviewer #2: Please see attached review.

Reviewer #3: The paper introduces ProteoBayes, a Bayesian framework for differential analysis that uses conjugate hierarchical Gaussian models to obtain closed-form posteriors for group means/variances, then performs inference by sampling from posterior distributions of mean differences. It extends naturally to a multivariate setting to account for inter-element correlations, argues that missing data should typically be ignored in univariate MAR settings, and proposes a multiple-imputation based approximation for the multivariate case. It also promotes probability-based, effect size centric decision criteria as more interpretable than p-values, and evaluates performance on synthetic and controlled spike-in proteomics datasets.

Pros for the proposed method:

1. Computationally tractable Bayesian inference using analytical prosteriors instead of using MCMC based approaches.

2. Robust uncertainty quantification using credible intervals and effect sizes instead of relying solely on p-values.

3. Natural treatment of missingness.

4. Multivariate extension that can capture within-protein peptide correlations.

Minor Comments:

1. The paper recommends to ignore missingness in the case of MAR, however other types of missingness is out of scope for the paper. It would however be interesting to see the robustness of the method in such scenarios through simulation studies.

2. It would be interesting to a senstivity analysis on the choice of prior for the method and how robust in the method to different choices of prior.

3. In the multivariate simulation study, it would be interesting to see how well the method performs when the sample size is small as computing the covariance matrix would be difficult.

**********

**Have the authors made all data and (if applicable) computational code underlying the findings in their manuscript fully available?**

The PLOS Data policy requires authors to make all data and code underlying the findings described in their manuscript fully available without restriction, with rare exception (please refer to the Data Availability Statement in the manuscript PDF file). The data and code should be provided as part of the manuscript or its supporting information, or deposited to a public repository. For example, in addition to summary statistics, the data points behind means, medians and variance measures should be available. If there are restrictions on publicly sharing data or code —e.g. participant privacy or use of data from a third party—those must be specified.

Reviewer #1: None

Reviewer #2: None

Reviewer #3: Yes

**********

PLOS authors have the option to publish the peer review history of their article (what does this mean?). If published, this will include your full peer review and any attached files.

If you choose “no”, your identity will remain anonymous but your review may still be made public.

**Do you want your identity to be public for this peer review?**  For information about this choice, including consent withdrawal, please see our Privacy Policy.

Reviewer #1: No

Reviewer #2: No

Reviewer #3: No

[NOTE: If reviewer comments were submitted as an attachment file, they will be attached to this email and accessible via the submission site. Please log into your account, locate the manuscript record, and check for the action link "View Attachments". If this link does not appear, there are no attachment files.]

**Figure resubmission:**

**Reproducibility:**

?>

Attachment Submitted filename: PLOS_Review_Final.pdf

# Author comment: Author response to Decision Letter 1

**DOI:** 10.1371/journal.pcbi.1014637.r002

28 Apr 2026

Attachment Submitted filename: Response_to_Reviewers.pdf

# Peer review: Decision Letter 1

**DOI:** 10.1371/journal.pcbi.1014637.r003

**Authors:** Ilya Ioshikhes

27 Jul 2026

Dear Dr. Chion,

We are pleased to inform you that your manuscript 'A Bayesian Framework for Multivariate Differential Analysis' has been provisionally accepted for publication in PLOS Computational Biology.

Before your manuscript can be formally accepted you will need to complete some formatting changes, which you will receive in a follow up email. A member of our team will be in touch with a set of requests.

Please note that your manuscript will not be scheduled for publication until you have made the required changes, so a swift response is appreciated.

IMPORTANT: The editorial review process is now complete. PLOS will only permit corrections to spelling, formatting or significant scientific errors from this point onwards. Requests for major changes, or any which affect the scientific understanding of your work, will cause delays to the publication date of your manuscript.

Should you, your institution's press office or the journal office choose to press release your paper, you will automatically be opted out of early publication. We ask that you notify us now if you or your institution is planning to press release the article. All press must be co-ordinated with PLOS.

Thank you again for supporting Open Access publishing; we are looking forward to publishing your work in PLOS Computational Biology.

Best regards,

Ilya Ioshikhes

Section Editor

PLOS Computational Biology

Ilya Ioshikhes

Section Editor

PLOS Computational Biology

***********************************************************

Reviewer's Responses to Questions

**Comments to the Authors:**

**Please note here if the review is uploaded as an attachment.**

Reviewer #1: The author has already answered my opinions very well. I have no more questions

Reviewer #2: The revised manuscript presents a compelling and computationally efficient Bayesian framework for differential analysis. The transition from standard p-values to probabilistic measures like the Total Variation Distance provides a highly intuitive and mathematically rigorous tool for biological decision making.

Your responses to previous reviewer concerns are thorough and structurally sound. Expanding the empirical comparisons to include proDA and MSqRob adds essential context that validates the performance of your method against modern standards. Furthermore, the new sensitivity analysis for hyperparameters successfully demonstrates the robustness of the conjugate model under various conditions.

The clarification regarding the Missing At Random assumption is particularly valuable. By explicitly distinguishing between contexts where missing data can be analytically integrated and Missing Not At Random contexts that require explicit modeling, you prevent future misuse of the computational framework.

The software package and accompanying data repositories meet the highest standards of transparency and reproducible research expected by the computational biology community. This manuscript represents a significant methodological contribution.

Reviewer #3: The authors have provided a thorough and generally satisfactory response to the previous reviewers’ comments. The revised manuscript now more clearly defines the scope and assumptions of the method, particularly regarding MAR versus MNAR missingness, the practical limitations of multivariate covariance estimation, condition-specific covariance matrices, and interpretation of the proposed posterior summaries. The authors have also broadened the methodological comparisons, improved the explanation of the algorithms, added runtime benchmarks, and incorporated a notation table, prior-sensitivity analyses, and an additional MNAR simulation in the Supporting Information. Although some methodological limitations remain, these are now adequately acknowledged. I therefore consider the main concerns to have been sufficiently addressed and recommend acceptance.

**********

**Have the authors made all data and (if applicable) computational code underlying the findings in their manuscript fully available?**

The PLOS Data policy requires authors to make all data and code underlying the findings described in their manuscript fully available without restriction, with rare exception (please refer to the Data Availability Statement in the manuscript PDF file). The data and code should be provided as part of the manuscript or its supporting information, or deposited to a public repository. For example, in addition to summary statistics, the data points behind means, medians and variance measures should be available. If there are restrictions on publicly sharing data or code —e.g. participant privacy or use of data from a third party—those must be specified.

Reviewer #1: None

Reviewer #2: Yes

Reviewer #3: Yes

**********

PLOS authors have the option to publish the peer review history of their article (what does this mean?). If published, this will include your full peer review and any attached files.

If you choose “no”, your identity will remain anonymous but your review may still be made public.

**Do you want your identity to be public for this peer review?**  For information about this choice, including consent withdrawal, please see our Privacy Policy.

Reviewer #1: No

Reviewer #2: No

Reviewer #3: No

# Editor report: Acceptance letter

**DOI:** 10.1371/journal.pcbi.1014637.r004

**Authors:** Ilya Ioshikhes

PCOMPBIOL-D-25-02643R1

A Bayesian Framework for Multivariate Differential Analysis

Dear Dr Chion,

I am pleased to inform you that your manuscript has been formally accepted for publication in PLOS Computational Biology. Your manuscript is now with our production department and you will be notified of the publication date in due course.

The corresponding author will soon be receiving a typeset proof for review, to ensure errors have not been introduced during production. Please review the PDF proof of your manuscript carefully, as this is the last chance to correct any errors. Please note that major changes, or those which affect the scientific understanding of the work, will likely cause delays to the publication date of your manuscript.

Soon after your final files are uploaded, unless you have opted out, the early version of your manuscript will be published online. The date of the early version will be your article's publication date. The final article will be published to the same URL, and all versions of the paper will be accessible to readers.

For Research, Software, and Methods articles, you will receive an invoice from PLOS for your publication fee after your manuscript has reached the completed accept phase. If you receive an email requesting payment before acceptance or for any other service, this may be a phishing scheme. Learn how to identify phishing emails and protect your accounts at https://explore.plos.org/phishing.

Thank you again for supporting PLOS Computational Biology and open-access publishing. We are looking forward to publishing your work!

With kind regards,

Janani Seenivasan

PLOS Computational Biology | Carlyle House, Carlyle Road, Cambridge CB4 3DN | United Kingdom ploscompbiol@plos.org | Phone +44 (0) 1223-442824 | ploscompbiol.org | @PLOSCompBiol
